# Transforming routine health data use in LMICs through modular, AI-supported automation: insights from Zimbabwe

**DOI:** 10.1093/oodh/oqag003

**Published:** 2026-01-30

**Authors:** Efison Dhodho, Kenneth Masiye, Forget Banda, Tafadzwa Bepe, Nqabutho Nyathi, Theonevus T Chinyanga

**Affiliations:** Organisation for Public Health Interventions and Development, OPHID, 96 West Road, Avondale West, Harare, Zimbabwe; Department of Global Health and Development, London School of Hygiene and Tropical Medicine, Keppel Street, London, WC1E 7HT, London, UK; Organisation for Public Health Interventions and Development, OPHID, 96 West Road, Avondale West, Harare, Zimbabwe; Organisation for Public Health Interventions and Development, OPHID, 96 West Road, Avondale West, Harare, Zimbabwe; Organisation for Public Health Interventions and Development, OPHID, 96 West Road, Avondale West, Harare, Zimbabwe; Organisation for Public Health Interventions and Development, OPHID, 96 West Road, Avondale West, Harare, Zimbabwe; Organisation for Public Health Interventions and Development, OPHID, 96 West Road, Avondale West, Harare, Zimbabwe

**Keywords:** modular automation, digital health, routine health data, health information systems, data use, artificial intelligence, LMICS, HIV programme

## Abstract

Health information systems (HIS) in Low- and Medium-Income Countries (LMICs) are often hindered by fragmented data flows, manual reporting processes and limited analytical capacity. These challenges compromise data quality, divert critical resources from patient care, delay reporting and limits the use of routine data for programme improvement. This descriptive case study documented the design, co-creation and rollout of the Organization for Public Health Interventions and Development Modular Data Intelligence Platform (OMDIP) across 15 districts in Zimbabwe. System performance and user experience were assessed through routine metrics, dashboards, supervision reports and user feedback collected between January 2023 and June 2024. The reporting of the intervention was guided by selected domains of the WHO mHealth Evidence Reporting and Assessment checklist. Development of the OMDIP began in May 2023. Additional modules were added: ReportAID AI enabled module for narrative synthesis, Data Diagnostic Module for error detection, the Data Analytics Platform for visual dashboards and the Data Export Request Listener for automated submission ready reports. These modules integrated with District Health Information System 2 and EHRs. Across 335 facilities supporting 345 000 clients, timely report submission improved from 27% to 100%, data-cleaning time decreased from 10.2 to 2.9 days, report preparation time dropped from 7 to under 2 days, and critical data errors were eliminated. OMDIP enhanced efficiency, quality and use of routine health data in Zimbabwe. Integrated with national systems and aligned with WHO digital health frameworks, it demonstrates a scalable model for strengthening data-driven decision-making and health system performance in LMICs.

## Introduction

In low- and middle-income countries (LMICs), effective health data management is a cornerstone of responsive and resilient health systems and a pathway towards universal health coverage. Robust health information systems (HIS) play a critical role in supporting evidence-based decision-making, facilitating resource allocation and monitoring programme performance (WHO, 2023). However, many LMICs especially in Sub-Saharan Africa continue to face longstanding challenges in strengthening their HIS, including fragmented reporting systems, manual data entry processes and limited human resource capacity to analyse and use data for timely action (Gloyd et al., 2016).

A well-functioning HIS is not merely a technical asset; it is a foundational socio-technical enabler for learning, accountability and improved population health outcomes. Evidence from large-scale reviews of digital health and routine information system implementations in LMICs consistently demonstrates that the performance of health systems is closely tied to how effectively routine data are produced, interpreted and used within everyday decision-making processes, rather than to the mere presence of digital tools alone (Braa & Sahay, 2012). Yet, LMICs frequently grapple with duplicated reporting burdens, and variable data quality, all of which hinder their ability to harness routine data for strategic decision-making (WHO, 2023). The reliance on paper-based tools and labour-intensive tallying further reduces the efficiency of health systems by overburdening frontline health workers who must divert time from service delivery to manage complex reporting requirements. Additionally, substantial resources are shifted from core program management and clinical care to support increasingly complex reporting needs as health systems pivot towards more data driven decision making (Siyam et al., 2021). The reporting itself is then unidirectional lacking feedback loops at multiple levels for efficient data utilization.

In response to these challenges, digital health innovations are increasingly being explored to streamline health data processes, improve data quality and promote timely insight generation. A major scoping review by (Lemma et al., 2020) had the main finding being that digital platforms have been shown to reduce inefficiencies and allow health systems to scale and adapt more rapidly. The WHO’s Global Strategy on Digital Health advocates for the development of interoperable, user-centred systems that not only integrate with existing digital infrastructure but also empower national stakeholders to use real-time data for planning and accountability (WHO, 2021).

## Background and problem statement

Zimbabwe’s Ministry of Health and Child Care (MoHCC) has progressively adopted digital transformation to improve access, quality and efficiency of health services. Guided by the *Zimbabwe Digital Health Strategy 2021–2025*, the country envisions ‘connected, accessible and low-cost, world-class health services’ built on interoperable and person-centered digital solutions (MOHCC, 2022). The National Health Information System is anchored on the District Health Information System 2 (DHIS2) platform, which serves as the primary national repository for routine data across public health programmes. Complementary systems include the Electronic Patient Monitoring System for HIV and TB, the Laboratory Information Management System and the Electronic Logistics Management Information System, all of which support routine reporting and supply-chain functions. (MOHCC, 2022). Zimbabwe’s national monitoring and evaluation (M&E) system remains a hybrid process combining both paper-based and digital workflows. Data capture begins at the facility level using standardized paper registers, which are then summarized into monthly reporting forms. These forms are manually collated, and data are extracted from the registers for submission to the district health office. District-level staff manually enter the information into the DHIS2, from which national reports are generated for program review and decision-making. However, data reporting for external stakeholders, such as donor agencies like PEPFAR, often requires the same data to be downloaded, cleaned and re-entered into separate reporting systems. Each of these manual transitions introduces opportunities for transcription errors, omissions and delays. The cumulative effect of multiple data handling points results in inefficiencies and frequent inconsistencies that require resource-intensive Data Quality Assessments and on-site data verification exercises to resolve. These systemic challenges underscore the need for automation and integrated data pipelines to improve accuracy, timeliness and overall confidence in routine health information (USAID Office of Inspector General [USAID OIG], 2022).

At the same time, Zimbabwe continues to experience a high burden of HIV, with an adult prevalence estimated at 11.3% and approximately1.3 million people living with HIV (MOHCC, 2020). Routine health data are central to tracking program performance, informing resource allocation and sustaining epidemic control efforts. Recognizing that traditional data collection and reporting methods were limiting responsiveness, the MoHCC, with support from implementing partners such as Organization for Public Health Interventions and Development (OPHID) has prioritized the modernization of data systems through digital platforms to automate reporting, enhance data quality and enable real-time decision support at national and subnational levels.

Within this national framework, OPHID has played a pivotal role as a technical partner in strengthening both public health service delivery and data systems, particularly within the HIV programme. The organization supports more than 335 public sector health facilities across 15 districts (out of the total 64 nationally), providing care to over 345 000 clients (25% of the 1.3 million people living with HIV on ART in Zimbabwe). The scale and complexity of service delivery generate a substantial reporting burden: each district produces monthly, quarterly and ad hoc reports from over 20 facilities, covering more than 20 indicators for diverse stakeholders including the MoHCC, PEPFAR and the Global Fund. Prior to the introduction of digital automation, these processes were largely manual, fragmented and error-prone, resulting in data duplication and significant reporting delays. Meeting these reporting demands require at least four full-time M&E officers per district diverting critical human resources from service delivery. National teams are left to manually synthesize 15 district reports, each exceeding 60 pages, within limited timeframes, making it difficult to extract actionable insights and provide critical feedback to health facilities. Variations in reporting formats and disaggregation requirements further increase workload, reduce consistency and delays programmatic responses.

To address these persistent inefficiencies, OPHID designed and implemented the OMDIP. This is a modular, interoperable, automation information system that streamlines data collection, cleaning, analysis and reporting. Grounded in principles of co-design, real-time feedback and alignment with national data standards, OMDIP represents a critical innovation toward strengthening routine data use, improving data quality and accelerating decision-making within Zimbabwe’s HIV programme.

### Aim

This case study aims to describe the design, development, deployment and early implementation outcomes of the OMDIP within Zimbabwe’s public health system, illustrating how a context-responsive and system-aligned digital solution can enhance routine data management, reporting and use in Zimbabwe and in other LMIC settings with comparable health system structures, data architectures and reporting constraints.

### Objectives

1) To describe how the modular digital platform was designed, developed and deployed to address routine data analysis, reporting and utilization bottlenecks within Zimbabwe’s HIV programme.
2) To assess how automation improved efficiency, reduced manual workload and enhanced data quality, completeness and timeliness across district and national levels.
3) To reflect on lessons learned and identify opportunities for sustainability, scale-up and adaptation to other programme areas within the national Health Information System (HIS) and broader application to other LMIC settings.

## Materials and methods

This study adopts an implementation-focused case study design to document the design, development, deployment and early implementation of the OMDIP within Zimbabwe’s public health system. The study draws on multiple quantitative and qualitative data sources collected between January 2023 and June 2024, coinciding with the development, phased rollout and full operationalization of OMDIP across fifteen districts in Zimbabwe. Data were drawn from routine programme reports, digital dashboards, supervision findings and structured user feedback to capture both system performance and user experience.

Quantitative data were primarily extracted from DATIM (the PEPFAR reporting platform) log reports, DHIS2 data-entry dashboards and OMDIP analytical modules, which record report completeness, timeliness and error detection. These datasets provided a consistent basis for assessing reporting efficiency and data concordance across systems. In addition, user activity logs from OMDIP modules offered objective measures of system utilisation, frequency of data entry and automation performance.

Qualitative data were sourced from several complementary channels, including quarterly report narratives prepared by district programme managers, supervision reports compiled during routine field visits and user feedback collected during monthly and quarterly report-writing sessions facilitated by OPHID and the MoHCC. Feedback from USAID and MoHCC technical teams was also reviewed to capture stakeholder perspectives on data quality, reporting timeliness and system usability. While the reports and feedback provided insight into perceived benefits and adoption, their potential for positive reporting bias was recognized and balanced through triangulation with supervision findings and routine performance data.

Key performance indicators focused on reporting timeliness, completeness and data quality, alongside measures of operational efficiency and user adoption. Data timeliness was defined as the proportion of facility- and district-level reports submitted to central level within prescribed deadlines, while completeness reflected the proportion of expected reports received each month. Data quality was evaluated using validation rules embedded within OMDIP and triangulated against DHIS2 and DATIM submissions. Efficiency was assessed by comparing the average number of days required for report preparation before and after OMDIP implementation. User adoption and engagement were inferred from system access logs, participation in report-writing sessions and qualitative accounts of workflow changes.

Quantitative indicators were analysed descriptively to assess trends in timeliness, completeness and efficiency. Qualitative material, including supervision notes, user reflections and narrative reports, was analysed using an implementation-focused case study analytic approach involving iterative review and triangulation across embedded data sources. This approach enabled the identification of recurring implementation patterns related to automation benefits, usability and data-use practices, rather than formal thematic coding. Triangulation across data types strengthened the credibility of findings and supported a comprehensive understanding of OMDIP’s role in enhancing routine health data quality and use.

The reporting of OMDIP was guided by selected elements of the WHO mHealth Evidence Reporting and Assessment (mERA) checklist, including technology platform, interoperability, intervention delivery and content, usability testing, user feedback, access, adoption inputs, limitations for delivery at scale, data security, replicability and alignment with national guidelines. The case study is organized using a digital health intervention lifecycle framework adapted from that utilized by Aleksandrenko et al. (2025) in their assessment of an mHealth (mobile health) deployment in Ukraine, enabling a phase-based analysis of implementation. This framework spans creation and design (ideation, co-design and modular development), deployment (district-level rollout and user onboarding) and maintenance (continuous iteration and adaptive learning).

## Results

### Development, implementation and phased scale up

**Figure 1:**
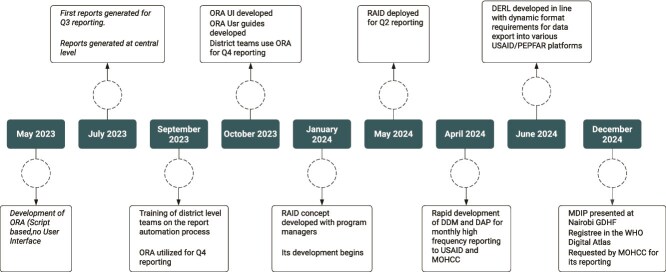
OMDIP modular development timelines.

Development of the OMDIP began in May 2023 with the piloting of the OPHID Report Automation (ORA) module in Bulilima District. Following positive results and peer-led demonstrations during program review meetings where all the 15 districts where in attendance, demand for the tool increased rapidly. ORA was subsequently expanded to six additional districts by July 2023 and achieved full scale-up across all 15 OPHID-supported districts by October 2023. (Fig. 1).

Building on the standardisation achieved through ORA, the need for efficient national report consolidation prompted development of the Report Aid (RAID) module in January 2024, incorporating an AI-supported model for automated narrative synthesis. Concurrently, additional modules were introduced and iteratively refined: Data Diagnostic Module (DDM) for automated error detection, Data Analytics Platform (DAP) for real-time trend analysis and Data Export Request Listener (DERL) for automated dataset formatting. We developed the platform using a co-design and co-production model that actively engaged end users, including nine data clerks, 15 district M&E officers and 10 programme leads. Co-design was operationalized through short feedback loops between frontline users and an embedded development team, rather than through direct end-user modification of system code. Users iteratively articulated emerging needs and constraints during routine use, which were translated into rapid system adaptations by locally embedded technical staff.

### Technical approach

We built the platform using open-source technologies, with Python and Rust handling most of the backend processing. We enabled data exchange through Extensible Markup Language, JavaScript Object Notation and Comma-Separated Values, ensuring seamless integration with other systems. For the user interface, we used Slint to develop modern, native desktop applications and Hypertext Markup Language, JavaScript and Cascading Style Sheets for web development. While earlier versions of the native client were developed using Qt (a cross-platform software development framework) and Python, we transitioned to a Rust + Slint stack to achieve better performance. Python continues to play a central role in data modelling and analytics.

Rather than connecting directly to databases, we designed the platform to interact with data via Application Programming Interfaces (APIs), using SQL Alchemy (a Python-based Object Relational Mapper) to abstract connections to underlying sources such as PostgreSQL and MySQL when necessary. For lightweight, on-the-fly querying, we use DuckDB as an in-process temporary database, ideal for rapid analytics without deploying a full database engine. On the data manipulation side, we relied on Pandas and Polars (via Python and Rust) for fast, flexible processing. These tools power everything from real-time insights to batch analytics. Finally, we deployed the system using a hybrid infrastructure, supporting both cloud-based and local machine environments. It is optimized for areas with intermittent internet access, offering full offline functionality with periodic synchronisation to ensure consistency and data integrity across all levels. This technology stack was intentionally selected to ensure low-cost deployment, minimal dependency on proprietary software, and high maintainability in LMIC environments, where infrastructure reliability and technical capacity may vary. The emphasis on open-source, modular and resource-efficient tools aligns with the WHO digital health principles promoting scalability, interoperability and sustainability in digital health solutions for public health programmes.

### Functional description of the platform modules

**Figure 2:**
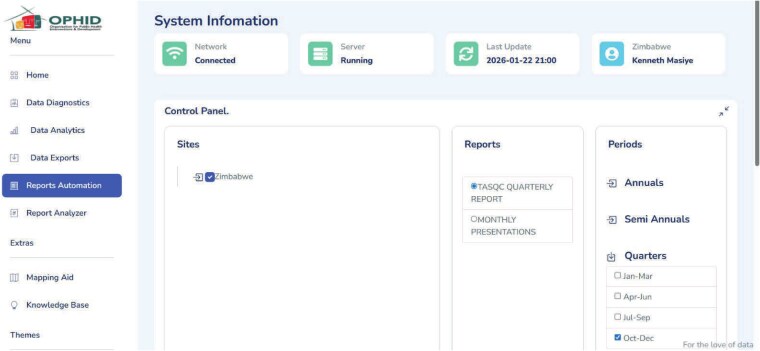
The OPHID data intelligence platform modules and user Interface.

The platform comprises five integrated modules (see Fig. 2), DDM, DAP, ORA, RAID and DERL. Each is designed to address key pain points in the health data lifecycle. Together, they operationalize the platform’s aim of improving the quality, efficiency and usability of routine programme data. The modules align with four core objectives: creating a user-centred modular system; reducing manual workload; improving data quality and timeliness; and generating actionable insights for programme improvement. Each is described in further details below.

### Data diagnostic module

**Figure 3:**
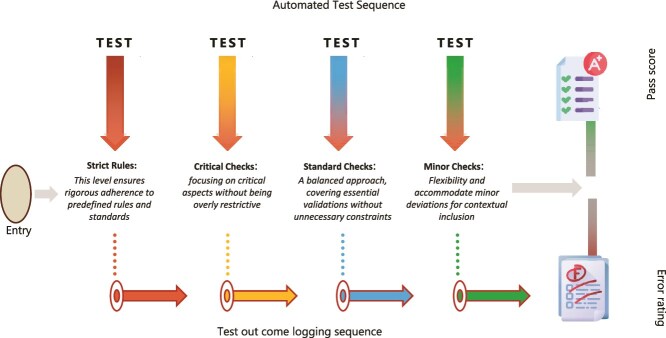
Schematic for workflow of the data diagnostic module (DDM), illustrating layered rule-based error detection.

The DDM was developed to address widespread, and recurring issues related to data quality. Historically, the identification of missing values, outliers and logic violations required manual review of large Excel files and on-site data verification exercises. These processes were not only time-consuming but also inconsistent and error prone. See Fig. 3 below.

### Data analytics platform

**Figure 4:**
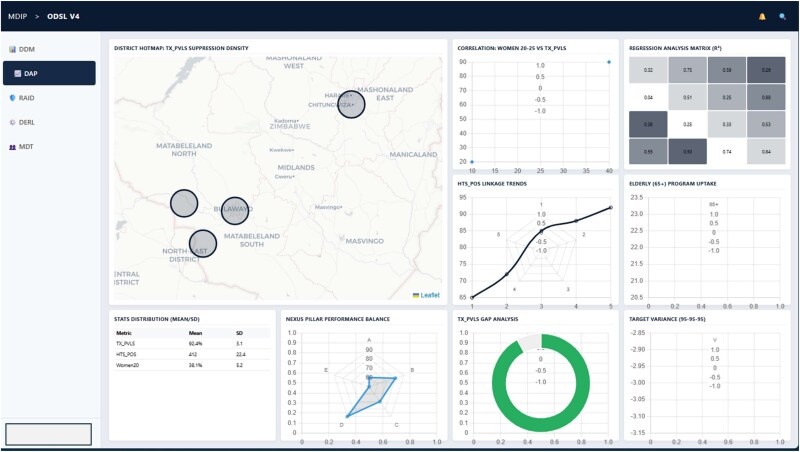
Screenshot of the data analytics platform (DAP), comparing expected and actual performance for key indicators.

DAP addresses the historical gap in the system’s capacity to produce timely, programmatically relevant insights. Previously, the detection of performance trends or correlations required time-intensive analysis by central M&E teams, often long after the reporting period had ended. See Fig. 4 below.

### OPHID report automation

**Figure 5:**
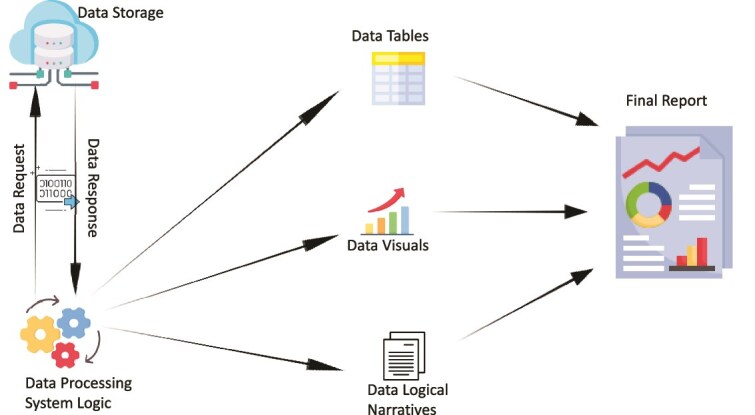
Process schematic for ORA showing automated report generation from DHIS2 data.

We developed the ORA module to address inefficiencies in manual report writing. Previously, M&E officers had to compile charts, embed visuals, into their Microsoft word reports manually, an intensive process often marked by delays and inconsistencies. See Fig. 5 below.

ORA automates this workflow by extracting data from DHIS2 and generating complete Microsoft narrative reports with standardized charts, indicator cascades and explanatory text pre-inserted. With a single click, users can produce quarterly reports that are visually consistent and aligned across all districts.

### Report AID

**Figure 6:**
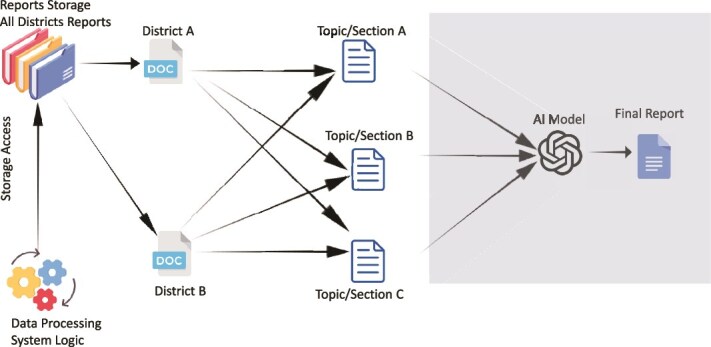
Architecture of RAID module using NLP to synthesize district-level narrative reports.

A natural language processing module, RAID (Fig. 6 below) was developed by OPHID to extract key statements from each section of the district reports and generate summarized versions of these reports.as needed.

The RAID module addresses the difficulty of synthesising qualitative content from lengthy district reports. Within it, we integrated a Natural Language Processing (NLP) model to automate the generation of narrative summaries from routine programme narratives coming from numerous health facilities. The model was developed using a Python-based transformer architecture built on the Bidirectional Encoder Representations from Transformers (BERT) framework, fine-tuned on a corpus of historical quarterly and annual programme reports produced by OPHID between 2018 and 2023. This training dataset included narrative interpretations, enabling the model to learn associations between quantitative trends and descriptive summaries. The AI system operates through a local API deployed within OMDIP’s analytics layer. When new data are submitted to the source folder, the RAID module is operated to extract the relevant indicators and generates a preliminary textual summarization with fuzzy matching for frequency of mention of an issue. This way the narrative output identifies key patterns, e.g. improvements or declines in specific indicators, anomalies or districts requiring follow-up, and presents them in concise, human-readable paragraphs of bullet points.

### Data export request listener

**Figure 7:**
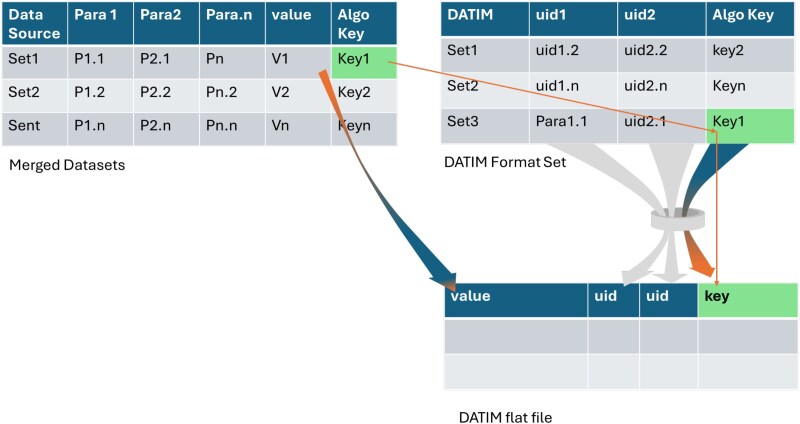
Algorithmic flow of the DERL, detailing rules-based export formatting.

DERL automates the transformation of aggregated data into standardized formats required by each stakeholder. It uses rule-based logic to apply appropriate age-sex disaggregations, funding mechanisms, and indicator categories, and generates validated submission files for each reporting stream. The data export request listener utilizes advanced algorithms to dynamically format data into any requested format, including specialized formats. The rules-based logic underpinning export formatting and validation within DERL is illustrated in Fig. 7 below.

It reduces errors in final reports, streamlines the export process and ensures timely submission of high-quality data to national and donor systems.

### Outcomes

#### Improved timeliness and efficiency of reporting

Prior to automation, only 4 of 15 districts consistently submitted reports to USAID by the required deadline (23rd of each month). Following implementation, all 15 districts submitted on time (report submission rose from timeliness improved from 27% to 100%). The average time required for data cleaning and error resolution decreased from 10.2 to 2.9 days per district. As one District M&E Officer reflected, *“Before, we were always playing catch-up. Now, by the 15th, we’re wrapping up, not still cleaning data.”*

#### Reduction in manual workload and improved reporting consistence

Utilization of ORA enabled the program teams to cut quarterly narrative report preparation time from 5–7 days to under at most 2 days by auto-generating charts, tables and narrative outlines. Report consistency improved from 35% to 100%, with all districts now using a harmonized format. As one district M&E officer put it*, ‘We now focus on what the data means, not how to compile it. That’s the biggest shift.’*

#### Enhanced data quality and standardisation

The DERL module automates dataset formatting, producing disaggregated reports for each stakeholder in the required format, eliminating the back-and-forth that previously occurred, sometimes up to three times, before acceptance. There was 100**%** reduction in critical errors in submitted datasets, including missing values, indicator mismatches and disaggregation inconsistencies. OPHID was cited by PEPFAR as a leader in report completeness and accuracy during quarterly all implementing partner meetings.

#### Use of data for programme learning and action

District and facility managers began initiating their own reviews, guided by visual dashboards and early warning alerts from the Data Analytics Platform. Over the four reporting cycles from Q3 2023 to Q4 2024, all 15 districts conducted mini-data reviews based on flagged anomalies, while 3 districts restructured outreach strategies in response to insights showing underperformance among adolescent girls and young women (AGYW). At least 6 districts began presenting dashboard-driven insights at provincial review meeting, something not previously seen. *‘For the first time, we feel like we own the data, not just collect it for someone else,’* shared a District Programme Manager. A USAID reviewer similarly observed, *‘Even the national quarterly summary now reads like a strategic report, not just pasted tables.’*

## Discussion

The implementation of the OPHID Data Intelligence Platform offers a compelling example of how context-responsive digital health innovations can enhance routine data systems in LMICs While much of the digital health literature has focused on patient-facing technologies and mobile applications, this case contributes critical insights into how systems-level platforms, when co-designed, strategically positioned and continuously adapted, can strengthen institutional performance, enable learning and build data cultures grounded in usability rather than compliance (Labrique et al., 2018). This discussion is guided by selected domains from the mERA checklist (Agarwal et al., 2016) and analysed through the Classification of digital interventions, services and applications in health (WHO, 2023). It seeks to explain the observed changes associated with OMDIP’s design, deployment and adaptive learning through that framework. The framework enables examination of the platform’s performance across three key phases: co-design and development, deployment and adoption and maintenance and adaptive learning.

### Design framework and development approach

The conceptualisation of OMDIP was rooted in a systems-thinking, bottom-up approach to digital health, drawing from international frameworks. In line with the World Health Organization’s Global Strategy on Digital Health (WHO, 2021), the platform was designed to address key limitations in fragmented and manual HIS. Theoretical underpinnings from modular information system architecture informed the structural design, allowing discrete functional units to be developed and deployed incrementally. This modularity provides flexibility, reduces systemic complexity and ensures the system can adapt to evolving programme needs without requiring full overhauls. Crucially, this design choice also facilitated seamless integration with existing national systems such as DHIS2 and facility-based EHRs which follow modular design, maintaining alignment with Zimbabwe’s broader digital health architecture (Labrique et al., 2018).

### Design and co-creation: usability, feedback and organisational alignment

Co-creation was foundational to OMDIP’s success and directly influenced adoption, usability, and sustainability. Each module was iteratively refined through weekly agile sprints that included programme managers, M&E officers, Strategic Information and Evaluation (SIE) managers and developers. This participatory design process aligns with mERA domains on usability testing, intervention delivery and user feedback, ensuring that system functionality was informed by operational realities rather than theoretical workflows. For example, design feedback from district M&E officers led to automated chart exports and batch-report generation in ORA, reducing report preparation time from five to seven days to under two days. Similarly, user-requested validation dashboards in DDM enabled near-real-time detection of data inconsistencies, which contributed to the observed 100% reduction in critical dataset errors.

User feedback during early piloting informed specific design changes such as automated chart exports in ORA and expanded error categories in DDM, enhancing relevance and ease of use. Peer-to-peer demonstrations and mentorship further accelerated adoption, reflecting the observation made by (Butler et al., 2020), that locally driven learning and ownership mechanisms are critical in digital health implementation. The institutional embedding of the systems developer within OPHID’s SIE department operationalized DevOps principles, enabling real-time problem-solving and cross-functional collaboration. This configuration mirrors findings by (Karunarathne et al., 2024), who identified management support (81%) and organisational culture (74%) as leading success factors for digital transformation initiatives.

### Deployment and adoption: interoperability, scalability and behavioural shifts

During the deployment phase, OMDIP’s modular architecture enabled phased expansion with minimal disruption to existing workflows. Integration with DHIS2, Open Data Kit, Microsoft Excel, Power BI and Electronic Health Records allowed seamless interoperability and avoided the creation of parallel systems, an essential mERA element. The design prioritized scalability and alignment with WHO guidance emphasising standards-based, interoperable and sustainable digital health infrastructure (WHO, 2025). This was done through consultative alignment with core MOHCC systems both in use already and under development.

Interoperability ensured that data entered once at the district level flowed automatically into consolidated dashboards, eliminating redundant manual reporting and accelerating submission timelines. This design innovation explains the improvement in on-time submissions, as delays from re-entry and format conversions were removed. Automation also allowed programme staff to focus on interpreting results rather than preparing them. The ORA and RAID modules together reduced narrative compilation time by over 70%, contributing to a documented shift in staff behaviour, from compliance-oriented reporting to data-driven decision-making. Tasks previously requiring 5–7 days, such as data cleaning, report formatting and narrative compilation were completed within hours. Moreover, peer-led demonstrations and rapid feedback loops supported an organic diffusion model in which early adopters influenced peers across districts reinforcing adoption and institutional buy-in.

### Contribution to the LMIC digital health Knowledge Base

The OMDIP case contributes to the LMIC digital health literature by illustrating how modular, automation-enabled platforms can strengthen routine HIS when embedded within existing organisational workflows and governance structures. Recent scoping and systematic reviews demonstrate that improvements in routine data quality and use in LMICs depend on socio-technical alignment; combining technical tools with workflow integration, user engagement and feedback loops, rather than digitisation alone (Hoxha et al., 2022; Lemma et al., 2020; Syed et al., 2023). OMDIP provides a concrete example of how co-designed automation can reduce reporting burden while enhancing data timeliness and use in a public-sector setting. It illustrates how automation, when co-created with users, enhances ownership, efficiency and decision-making (Butler et al., 2020). The platform’s recognition by national programmes, including requests from the TB and laboratory divisions for adaptation and integration, further confirms its institutional relevance. These expansion requests reflect alignment with WHO’s recommendation that sustainability and impact in digital health depend on integration with national systems, adherence to data standards and flexibility to serve multiple programme areas (Agarwal et al., 2016; WHO, 2021).

Consistent with broader digital health synthesis evidence, this case highlights that sustainability and scale are enabled by modularity, interoperability and institutional embedding, rather than bespoke or stand-alone systems (Agarwal et al., 2016; WHO, 2021). Scalability is therefore framed not as geographic expansion alone, but as routinisation and adaptive integration within national reporting ecosystems, conditions relevant to other LMIC settings facing similar reporting burdens and system constraints. By foregrounding these mechanisms, the OMDIP case contributes to the LMIC digital health knowledge base by articulating how modular, system-aligned automation can support the transition from episodic reporting to more responsive, learning-oriented use of routine health data in settings with comparable reporting burdens and system constraints.

## Lessons learned

The deployment of the OPHID Data Intelligence Platform yielded several important lessons for digital health implementers and policy stakeholders in LMICs:

### Co-creation strengthens adoption and usability (mERA: Usability testing, user feedback, adoption inputs)

Engaging end users, particularly district M&E officers, programme managers and SIE teams, throughout the design and testing phases proved central to adoption and usability. Regular sprint-based feedback cycles allowed frontline users to refine functionality, such as adding automated chart exports in ORA and expanding validation categories in DDM. This participatory approach addressed usability and relevance early, aligning system design with actual workflows and cognitive load. It also operationalized *adoption inputs* by embedding local champions within each pilot district, ensuring peer-to-peer diffusion and sustained engagement. These processes validate evidence that user-led co-creation enhances ownership, trust and long-term use of digital systems (Braa & Sahay, 2012; Labrique et al., 2018).

### Organisational integration enables feasibility, interoperability and sustainability (mERA: Technology platform, interoperability, feasibility)

OMDIP’s institutional success was underpinned by a deliberate organisational design that integrated the systems developer within OPHID’s SIE directorate. This arrangement allowed for real-time feedback loops between programme staff and technical teams, embodying the DevOps principle of continuous integration. Often the disconnect between developers and implementers and organizational resources limits the seamlessness needed for co-creative and iterative development. Interoperability was achieved by aligning OMDIP with existing and in use national systems, including DHIS2, ODK, Power BI and EHRs, thereby avoiding redundant data entry or parallel reporting. This experience affirms global evidence that digital systems achieve scale and resilience when technically interoperable and institutionally embedded within existing health information structures (Karunarathne et al., 2024).

### Demonstrated value accelerates diffusion and replicability (mERA: Scalability, replicability, system maturity)

The measurable gains in efficiency, data quality and reporting consistency led to unsolicited requests from national programmes (including the TB and laboratory divisions) for adaptation of OMDIP’s modules. Such organic diffusion reflects high *system maturity* and *scalability*, driven by visible value to end users and institutional stakeholders. In mERA terms, the intervention demonstrated strong replicability because it was modular, standards-aligned and grounded in user needs. When digital tools demonstrably solve operational challenges and yield quantifiable time savings, they attract demand and become self-propagating echoing findings by that sustainability in digital health is a function of perceived value and integration within national systems (WHO, 2021).

## Conclusion

The OPHID Data Intelligence Platform demonstrates how locally grounded, user-centred digital innovations can strengthen HIS in LMICs. Its modular architecture and automation improved reporting timeliness, reduced workload, enhanced data quality, and enabled routine data to be used for continuous learning and action. Rather than replacing existing infrastructure, OMDIP constructively layered onto national systems such as DHIS2 and the MoHCC monitoring and evaluation framework, ensuring institutional alignment and interoperability.

Sustainability has been reinforced through MoHCC ownership of the platform’s governance, integration into national reporting workflows and adoption of OMDIP’s architecture for other programme areas. The platform has been licenced as Open Source using GLPv3 and is on course to being fully managed by MOHCC as part of its systems. The platform’s mostly open-source infrastructure and adherence to the Zimbabwe Cyber and Data Protection Act further ensure long-term functionality and data sovereignty. Unsolicited requests from the tuberculosis, laboratory and health information units illustrate growing cross-programmatic demand and institutional confidence, indicators of sustainability and national integration beyond donor cycles.

As LMICs continue to confront rising data demands with constrained resources, OPHID’s experience provides a blueprint for scalable, interoperable and user-led digital transformation that strengthens decision-making at every level of the health system. The platform’s design and outcomes align closely with the WHO guideline. Recommendations on digital interventions for health system strengthening (WHO, 2019) and advance the vision of Sustainable Development Goal 3 (Good Health and Well-being) by transforming routine health data into actionable intelligence for improved system performance and public health outcomes.

## Limitations and future research

This study presents an implementation-focused descriptive case study rather than a formal impact evaluation; as such, causal inferences regarding effectiveness cannot be drawn. Quantitative analyses were limited to routinely collected programme data over an 18-month period and focused on HIV programmes within OPHID-supported districts, which may limit generalisability to other health areas or system contexts. While qualitative feedback provided important implementation insights, it was derived from routine documentation, supervision reports and programme narratives rather than from independently conducted structured interviews and may therefore be subject to positive reporting bias.

Future research should complement this case study with mixed-methods implementation studies that incorporate structured qualitative inquiry and economic evaluation to assess cost-effectiveness, user experience and scalability. In addition, longitudinal assessment of OMDIP’s sustainability and institutionalisation within national digital health policy and governance frameworks will be important to inform responsible scale-up and adaptation in Zimbabwe and in other LMIC settings with comparable health system structures and reporting constraints.

## Security architecture, data governance and legal compliance

The OMDIP was developed and deployed in strict adherence to Zimbabwe’s Cyber and Data Protection Act [Chapter 12:07] (2021), which mandates responsible collection, processing and storage of personal and health-related data. All data handled within OMDIP are encrypted both in transit and at rest using Advanced Encryption Standard (AES-256) protocols to safeguard confidentiality and integrity. Access to the platform is restricted through multi-level user authentication, role-based permissions and session-based access controls that align with the MoHCC data governance framework.

Data exchange between OMDIP and external systems such as DHIS2, DATIM and partner databases occurs exclusively over secure Hypertext Transfer Protocol Secure channels through authenticated APIs. Data logs, audit trails and user activity records are maintained within the system to ensure traceability and accountability at every level.

OPHID, as a licensed Data Controller under the Zimbabwe Cyber and Data Protection Act, retains custodianship of system-level data management in coordination with MoHCC. All analytical and operational data generated within OMDIP are stored on secure servers located within Zimbabwe, with periodic encrypted backups hosted on redundant cloud environments approved by MoHCC. Access to these backups and live datasets is limited to authorized personnel bound by institutional data-use agreements and confidentiality clauses

## Data Availability

The source code and related technical artefacts for the OPHID Data Intelligence Platform are not yet publicly available due to ongoing licensing and institutional governance processes. OPHID is in the process of releasing the core modules under a GNU General Public License v3 (GPL v3), with public access anticipated upon completion of legal and documentation procedures. In the interim, high-level architectural documentation and module descriptions are available upon reasonable request. A demo version of the platform is also available for academic or non-commercial walkthroughs. For access to these materials, please contact the corresponding author.
